# Comparative preclinical pharmacokinetics study of 3,3′-diindolylmethane formulations: is personalized treatment and targeted chemoprevention in the horizon?

**DOI:** 10.1186/1878-5085-4-25

**Published:** 2013-12-10

**Authors:** Mikhail Paltsev, Vsevolod Kiselev, Ekaterina Muyzhnek, Vadim Drukh, Igor Kuznetsov, Olga Pchelintseva

**Affiliations:** 1 National Research Centre (NRC “Kurchatov Institute”), 1, Akademika Kurchatova Pl., Moscow 123182, Russia; 2 Peoples’ Friendship University of Russia, Miklukho-Maklaya St., 6, Moscow 117198, Russia; 3 ZAO “MiraxBioPharma”, 12 Kutuzovsky av., Moscow 121248, Russia; 4 Moscow State Medical Stomatological University (MGMSU), Delegatskaya St., 2/1, Moscow 127473, Russia

**Keywords:** 3, 3′-Diindolylmethane, Preclinical trials, Pharmacokinetics, Bioavailability, Molecularly targeted treatment, Targeted prevention, Personalized medicine, Breast cancer, *BRCA1*

## Abstract

**Background:**

3,3′-Diindolylmethane (DIM) is known as an agent of natural origin that provides protection against different cancers due to the broad spectrum of its biological activities *in vivo*. However, this substance has a very poor biodistribution and absorption in animal tissues. This preclinical trial was conducted to evaluate the pharmacokinetics and bioavailability of various DIM formulations in animal model.

**Materials and methods:**

The pharmacokinetic parameters of one crystalline DIM formulation and one liquid DIM formulation (oil solution) compared to non-formulated crystalline DIM (control) were tested in 200 rats. The formulations were orally administered to animals by gavage at doses of 200 mg/kg per DIM (crystalline DIM formulation and non-formulated crystalline DIM) and 0.1 mg/kg per DIM (DIM in oil solution). DIM plasma elimination was measured using HPLC method; after that, the area under the curve (AUC), relative bioavailability, and absolute bioavailability were estimated for two formulations in relation to non-formulated crystalline DIM.

**Results and conclusion:**

The highest bioavailability was achieved by administering liquid DIM (oil solution), containing cod liver oil and polysorbate. The level of DIM in rat blood plasma was about fivefold higher, though the 2,000-fold lower dose was administered compared to crystalline DIM forms. The novel pharmacological DIM substance with high bioavailability may be considered as a promising targeted antitumor chemopreventive agent. It could be used to prevent breast and ovarian cancer development in patients with heterozygous inherited and sporadic *BRCA1* gene mutations. Further preclinical and clinical trials are needed to prove this concept.

## Overview

At the present time, personalized medicine, focused on the individual approach to treatment, receives a powerful developmental impetus. This innovative trend improves management of diseases and increases the effectiveness of therapy by monitoring the specific clinical characteristics and molecular markers. Particularly, the personal genetic analysis for identification of mutations associated with different pathological disorders helps diagnose disease more accurately, predict risks, and choose the appropriate treatment for a particular groups of patients.

According to the numerous experimental and clinical data, 3,3′-diindolylmethane (DIM) is considered as a new chemopreventive agent in oncology. DIM is the major *in vivo* metabolite of indole-3-carbinol (I3C)—a phytochemical found in cruciferous vegetables. DIM is acid stable and is detected in the bloodstream after oral intake of I3C or DIM [1,2]. It is well known that the anticancer action of these substances of natural origin is directed to modulation of multiple components of cancer cell cycle regulation and survival including Akt/NF-κB signaling, cyclin-dependent kinase activities, caspase activation, estrogen receptor signaling, and estrogen metabolism [3-6].

As has been shown by Bradlow et al., the positive alterations of estradiol metabolism have been determined in different strains of mice with spontaneous mammary tumors that consumed I3C (*in vivo* precursor of DIM) at doses ranging from 34 to 700 mg/kg/day [7]. In other studies, the hydroxylation level of ‘good’ estrogen metabolite (2-hydroxy-estrone) was significantly increased in wild-type mice and transgenic mice receiving DIM at a dose of 300 mg/kg [8]. The same results were received in a pilot clinical study conducted in postmenopausal women with a history of early-stage breast cancer [9]. DIM also inhibits invasion of cancer cells and tumor neoangiogenesis [3]. We have recently reported that DIM is a selective and potent inhibitor of high tumorigenic cancer stem cells, or tumor-initiating cells [10].

Thus, due to the fact that DIM inhibits many harmful processes in early stages of carcinogenesis, this substance may be successfully used in cancer prevention in high risk groups of patients. Besides that, it can be considered as a novel approach of antitumor therapy in personalized medicine.

One of the established molecular targets of DIM is the breast cancer susceptibility 1 (*BRCA1*) gene. The prevalence of *BRCA1/2* mutation carriers in general population is around 0.2%, and about 5%–10% of all breast cancers and 10%–15% of ovarian cancer cases can be attributed to this risk factor [11]. In normal breast cells, tumor suppressor BRCA1 protein encoded by the *BRCA1* gene inhibits many carcinogenic processes including regulation of cell cycle progression, DNA damage signaling and repair, maintenance of genomic integrity, and the regulation of various transcriptional pathways, particularly the transcription of ERα gene and its downstream estrogen responsive genes [12]. When *BRCA1* gene is mutated, ERα is activated at low estrogen concentrations. As a result, high expression of estrogen responsive genes leads to uncontrolled cell growth. DIM upregulates the expression of gene/protein *BRCA1*, which inhibits ERα signaling [13,14]. Study reported by Fan et al. showed that low doses of DIM (1 μM) could stimulate *BRCA1* signaling and expression in breast and prostate cancer cells [15]. It should be mentioned that some other interesting data published in the previous years approve the ability of DIM to stimulate *BRCA1* expression in non-cancer diseases such as inflammatory bowel diseases and heart failures caused by oxidative stress [16,17].

According to the two-hit carcinogenesis model, the ‘point of no return’ is the loss of heterozygosity, i.e., the second mutation in the healthy (not damaged) gene allele in somatic cell after the first inherited mutation in germ cell [18]. So, the most acceptable chemoprevention strategy for patients with *BRCA1* (and other oncoprotective genes) mutation could be early pharmacogenetic correction, directed to genome stabilization. So, DIM may prevent breast cancer development in patients with heterozygous inherited and sporadic *BRCA1* gene mutations. Translation of this approach ‘from bench to bedside’ is the aim of the given stage of our work.

As we can conclude that *BRCA1* expression stimulation by DIM is well demonstrated *in vitro*, the next necessary stage should be a demonstration of this effect *in vivo* and then translate it into the disease response [19]. But, nowadays, *one of the main obstacles for investigators working with this potential pharmacological agent is an extremely low DIM bioavailability in vivo*. DIM usually shows low solubility in physiological liquids and possess very limited ability to penetrate through barrier membranes. Moreover, an ability of this compound to bind with plasma proteins and to be involved in various non-specific interactions in blood flow was shown. These facts significantly reduce the efficiency of delivery of DIM to a focus of disease.

It is well known that the drug pharmacokinetic study in animals is a necessary and obligatory step for estimation of its biodistribution in tissues. These investigations are also very important for the development of new DIM-based formulations with a high bioavailability [1,2,20,21].

We have developed two different DIM-based pharmaceutical compositions with potentially enhanced bioavailability of the active substance. In this study, DIM bioavailability of one crystalline formulation and one liquid formulation [21] was estimated and compared to non-formulated individual DIM substance. Additionally, comparative pharmacokinetic data after oral administration by gavage of various DIM formulations were estimated.

## Methods

### Reagents

The crystalline DIM without any additional agents was used as a pure non-formulated DIM (substance N1, powder). Crystalline formulation on the basis of DIM (substance N2, powder) consisted of lactose, microcrystalline cellulose, sodium starch glycolate, kollidon, starch, and magnesium stearate. Liquid formulation based on DIM (substance N3) was prepared according to special patented technology [22]. Substance N3 consisted of DIM and two components (cod liver oil and polysorbate) that provide increased solubility and bioavailability of DIM. All reagents were from Sigma-Aldrich (St. Louis, MO, USA). The following reagents and organic solvents were used for high-performance liquid chromatography (HPLC) analysis and previous extraction of active substances from blood plasma samples: acetonitrile, ethyl acetate, sodium dodecyl sulfate, potassium hydrogen phosphate, phosphoric acid (all reagents were from Sigma-Aldrich).

### Animals

The studies were carried out using 144 female Sprague–Dawley rats (0.22 ± 0.02 kg) which were purchased from Charles River, Inc. (Wilmington, MA, USA). Forty-eight rats were used to define the distribution of two novel DIM formulations and one control crystalline DIM substance (48 × 3 = 144 rats). The animals were housed in standard cages, given *ad libitum* access to food and water, and maintained on a 12:12 h light/dark cycle. The length of quarantine (acclimatization period) was 14 days. During this time, daily inspections of each animal (behavior and general condition) were undertaken. All rat procedures were approved by the Animal Care and Use Committees, and performed in accordance with institutional politics.

### Animal dosing and sampling

Samples of substance N1 (control) and substance N2 were administered to rats by atraumatic gavage at a dose of 200 mg/kg, substance N3—at a dose of 0.1 mg/kg—in the morning (fasting). Mother suspension based on starch mucus *ex tempore* (20 mg/200 μL) was prepared for the administration of substances N1 and N2. Dosing was carried out by volume of mother suspension, depending on the weight of each animal (on average, 200 mg/kg). Cod liver oil solution of DIM with polysorbate was used as a solvent for administration of substance N3 (on average, 200 μL/animal).

The animals received standard diet 2 h after the beginning of experiment. After various time intervals (0.25, 0.5, 1.0, 2.0, 4.0, 6.0, 8.0, and 12.0 h) post-dosing, the blood samples were collected. The rats were anesthetized by general inhalation of isoflurane (Sigma-Aldrich). The blood samples were collected from the jugular vein with heparinized tube and kept immediately in ice for 5 to 10 min. Then, the blood samples were immediately centrifuged, and the plasma was separated. The plasma samples were immediately frozen in dry ice and stored at -20°C until further analysis.

### Sample preparation for HPLC analysis

Before analysis, HPLC analytical procedure was validated according to the FDA procedures [23]. Five milliliters of ethyl acetate has been added in all blood plasma samples (1 mL). The samples were shaken for 2 min and then centrifuged at 4,000 rpm for 10 min. The supernatants were separated. The organic phase was evaporated in the stream of nitrogen at 50°C, until dryness. The dried samples were dissolved in 200 μL of acetonitrile and then diluted with mobile phase. After that, the aliquots were injected into HPLC for analysis.

### Sample extraction and analysis

The quantity of DIM in the blood plasma samples was measured by HPLC method using a liquid chromatograph (LC-20 Prominence, Beckman Coulter Inc., Brea, CA, USA) with UV detector (230 nm) and SPD-M20A diode array (Shimadzu, Japan).

The HPLC conditions were as follow: column Ascentis^®^ C_18_ (25 cm × 4.6 mm × 5.0 μm, Bellevue, WA, USA), column temperature 22°C–24°C, flow rate 1.0 mL/min, injection volume 5.0 μL. The mobile phase (1 L) comprised 70% of acetonitrile, 30% of aqueous buffer containing 6 mg of sodium dodecyl sulfate, 0.6 mg K_2_HPO_4_ and added with H_3_PO_4_ until pH 5.0, and the UV detection is at *λ* = 230 nm. The mobile phase was degassed and filtered before HPLC analysis. The retention time of DIM under these conditions was 5.07 min. The minimum sensitivity of the analytical method was estimated as 0.03 μg/mL.

The plasma DIM concentrations were calculated from the area under the peak (AUP) by using calibration curve. The areas under the curves (AUC) were calculated by trapezoidal method. The relative bioavailability (%) was calculated as a ratio of AUC (the time period from 0 to *∞*) for the investigated substance (AUC_Substance_) to the AUC for the control substance N1 (AUC_Substance N1_). Change in relative bioavailability (%) was calculated as a ratio of (AUC_Substance_ - AUC_Substance N1_) to AUC_Substance N1_. Absolute bioavailability was calculated based on the assumption that plasma and average tissue DIM concentrations are the same and taking into account the animal mass and volume of blood.

### Statistical analysis

The experimental data were treated statistically using Systat program package (Systat Software, Inc., Chicago, IL, USA), and the arithmetic mean (average) and standard deviation of the mean (SDM) were calculated. The following pharmacokinetic parameters were estimated using the WinNonLin program (Pharsight Corporation, Mountain View, California USA) by the model-independent method: time to reach maximum plasma concentration, maximum plasma concentration, AUC, mean residence time (MRT), volume of distribution (Vss), and elimination half-life (Т_1/2_). Plasma DIM levels below the limit of quantitation were set to 0.

## Results

**Figure 1:**
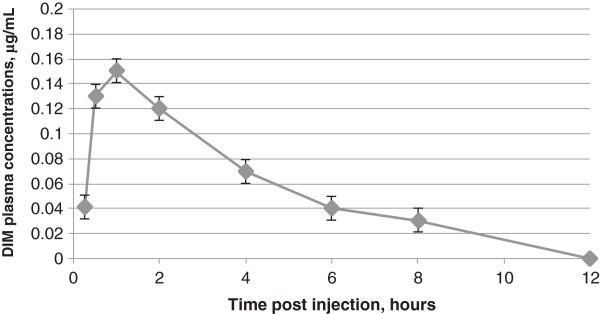
**Averaged dynamics of the DIM blood plasma elimination for substance N1 (non-formulated crystalline DIM).** Determined after oral administration to female Sprague–Dawley rats by gavage at a dose of 200.0 mg/kg/DIM.

DIM began to be determined in the systemic circulation 15 min after administration to the experimental animals of the control substance N1 (non-formulated crystalline DIM) at a dose of 200.0 mg/kg/DIM (Figure 1). The maximum plasma DIM concentration (*C*_max_) achieved at 1 h after administration was about 0.15 μg/mL (0.15 ± 0.01 μg/mL). Hereafter, DIM concentration started falling gradually, to a minimum (less than 0.03 μg/mL) at 12 h after administration. The subsequent decrease of plasma DIM concentration was characterized by T_1/2_ about 2 h (2.11 ± 0.04 h). MRT was about 4.5 h (4.46 ± 0.16 h). Vss was about 1,300 L/kg. The individual variability was moderate: CV ranged from 8% to 22%.

**Figure 2:**
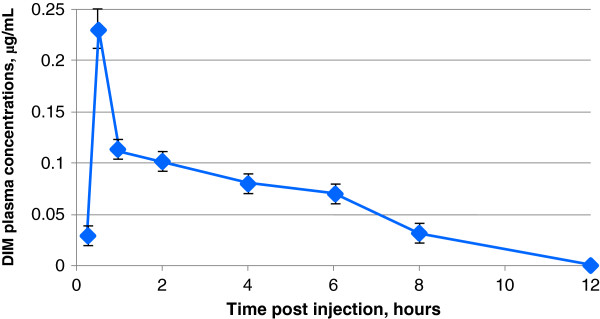
**Averaged dynamics of the DIM blood plasma elimination for substance N2 (crystalline form).** Determined after oral administration to female Sprague–Dawley rats by gavage at a dose of 200.0 mg/kg per DIM.

In the experiments with substance N2, DIM also began to be determined in the systemic circulation 15 min after administration at a dose of 200.0 mg/kg per DIM. *C*_max_ observed at 0.5 h after administration was 0.23 μg/mL (0.23 ± 0.01 μg/mL). Hereafter, as in the previous case, DIM concentration began falling gradually, to a minimum (less than 0.03 μg/mL) 12 h after administration (Figure 2). The subsequent decrease of plasma DIM concentration was characterized by T_1/2_ about 3 h (3.04 ± 0.05 h). MRT was about 5.2 h (5.19 ± 0.05 h). Vss was about 1,400 L/kg. The individual variability was moderate: CV ranged from 5% to 21%.

**Figure 3:**
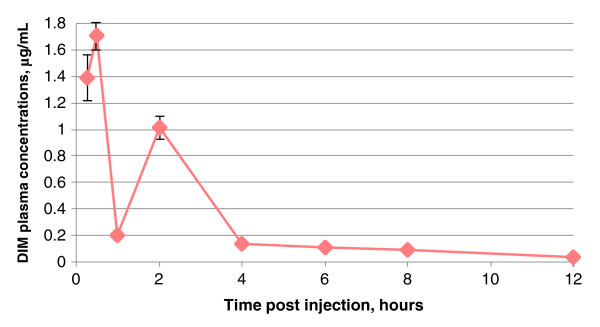
**Averaged dynamics of the (DIM) blood plasma elimination for substance N3 (liquid form).** Determined after oral administration to female Sprague–Dawley rats by gavage at a dose of 0.10 mg/kg per DIM. The value of standard deviation of the mean was less than or equal to 0.03 at the time of 1, 4, 6, 8, and 12 h (not shown).

After oral administration of the liquid DIM formulation (substance N3) by gavage, DIM also began to be determined in the systemic circulation 15 min after administration. However, *C*_max_ observed at 0.5 h after administration was 1.71 μg/mL (1.71 ± 0.05 μg/mL). The second peak of DIM concentration observed at 2 h after administration was 1.01 μg/mL (1.01 ± 0.04 μg/mL) (Figure 3). In should be pointed out that the real nature of the dual peak on the concentration-time curve is not clear yet and requires further study. We consider it necessary to emphasize that the dose of substance N3 was 0.10 mg/kg/DIM, i.e., it was 2,000 times less than the administered dose of substance N2 and control substance N1. Hereafter, as in the previous case, DIM concentration began falling gradually, to a minimum (about 0.03 μg/mL) 12 h after administration. The subsequent decrease of plasma DIM concentration was characterized by T_1/2_ about 3.8 h (3.75 ± 0.31 h). MRT was about 5.6 h (5.55 ± 0.41 h). Vss was approximately of 130 mL/kg. Individual variability was moderate: CV ranged from 5% to 20%.

In addition to the main pharmacokinetic parameters, relative DIM bioavailability, change in the relative bioavailability, and absolute DIM bioavailability for each substance have been estimated (Table 1).

**Table 1:** Relative bioavailability of different DIM formulations

**Substance number**	**AUC, μg·h/mL (mean** ± **SDM)**	**Relative bioavailability**	**Change in the relative bioavailability (%)**	**Absolute bioavailability (%)**
1	0.71 ± 0.04	-	-	0.006
2	0.78 ± 0.02	1.10	9.86	0.009
3	4.42 ± 0.55	6.23	522.54	98

The values were determined after oral administration of DIM to female Sprague–Dawley rats by gavage in relation to substance N1 (control).

It was established experimentally by other authors that blood plasma DIM concentrations must be significantly more than 200 ng/mL to achieve the therapeutic effect. Insufficient solubility and bioavailability of crystalline DIM formulations hamper the effective therapeutic DIM concentrations (concentrations which are effective in *in vitro* experiments) in target tissues and organs to be reached. As it was shown in our study, the highest bioavailability was achieved by administering liquid DIM formulation (substance N3) that is the oil solution containing polysorbate. Neither non-formulated crystalline DIM (substance 1) nor crystalline DIM formulation (substance 2) showed any significant bioavailability of active component which is necessary for therapeutic effects of DIM.

It can be assumed that in the case of substance N3, the high DIM concentration in peripheral blood was achieved due to the following facts. As a part of the new liquid formulation created by us on the basis of modern technological solution, DIM is in the dissolved state (oil solution), so it possesses an ability to enter quickly into the bloodstream and target organs. The auxiliary components of the organic origin in certain proportions in substance N3 not only dramatically enhanced DIM bioavailability but also increased drug stability during the storage. It was established that substance N3 is stable under acidic conditions and provides virtually 100% bioavailability in animal model.

## Conclusion

The crystalline DIM formulations (substance N2 and control substance N1) did not reveal high concentrations of active component (DIM) in blood plasma after oral administration by gavage to animals. At the same time, fivefold higher concentration of DIM was observed in blood plasma of rats who received the 2,000-fold lower dose of liquid DIM formulation compared to that of crystalline form and non-formulated crystalline DIM. Administration of liquid DIM formulation to animals resulted in achievement of physiological DIM concentrations necessary for increase of *BRCA1* gene/protein expression *in vitro*. Application of the new pharmacological liquid DIM substance with high bioavailability (substance N3) may be considered the perspective approach of effective personalized treatment and breast cancer prevention in patients of high-risk groups, particularly in women with *BRCA1* gene mutation. The data make this pharmaceutical formulation a priority candidate for further development.

## Expert recommendations

Hereditary breast cancer is usually detected in women with family history of breast cancer that was observed among first-degree relatives (mother, sister, and daughter) and is caused by mutations in tumor suppressor genes *BRCA1* and *BRCA2* in germinal cells. It is known that for women who have inherited a mutant allele of one of the *BRCA* genes from one parent, the likelihood of developing breast cancer during their lifetime is 80%–90% [24]. As a rule, such a tumor develops at a young age (*BRCA1*-related, at 35–39 years old; *BRCA2-*related, at 43–54 years old) and characterized by hormone-independent growth, high rate of development in the opposite gland, high degree of malignancy, tendency to relapse, and worse prognosis [25]. The most common option of treatment for these women is bilateral mastectomy, but this does not guarantee that cancer will not develop in other organs such as ovary. So, personalized pharmacogenetic chemoprevention by DIM-based pharmaceutical preparations could become a better alternative for them.

**Figure 4:**
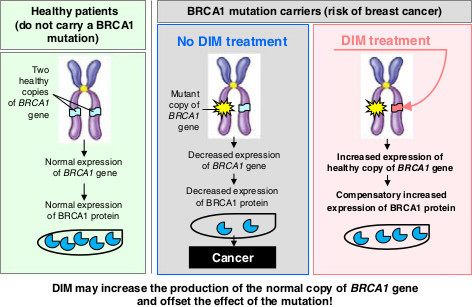
***BRCA1 *gene.** This gene is a proposed molecular target of DIM in personalized chemoprevention of breast cancer by high bioavailability DIM-based formulation.

Interest in the role of *BRCA* genes/proteins increased particularly in connection with recently discovered fact of their possible involvement in the development of non-hereditary (sporadic) cancer. According to preliminary data, loss of heterozygosity of the *BRCA1* gene is observed in more than half of the cases of sporadic breast cancer and ovarian cancer [26,27]. For these patients, enhancement of the *BRCA1* gene function by targeted DIM impact may be particularly essential for prevention of cancer progression. It can be assumed that DIM influence will take place by means of DIM-mediated increased production of the normal copy of *BRCA1* gene which will offset the effect of the *BRCA1* mutation (Figure 4).

We believe that our research will promote the development of a very interesting approach in personalized cancer chemoprevention. Up to this moment, there are no drugs with such a potential for public health care. The common problem for anticancer drug development is high toxicity of new candidates. DIM is considered as a relatively safe substance [28-31]. So, we can suppose that our liquid DIM formulation may be very promising for drug development, especially for the benefit of individual patients and for health care in general.

As we know now, DIM is the only *BRCA1* stimulating agent on clinical trial stage [32], but that trial is conducted with DIM formulation having relatively low bioavailability. Concerning personalized treatment safety of our liquid DIM pharmaceutical composition, we know that the only possible limitation of its use is the specific DIM toxicity in Crohn’s disease patients. There is evidence that the risk of *Escherichia coli* penetration into the gut wall may be increased in the presence of polysorbate (a component of liquid DIM formulation) in these patients [33]. However, on the other hand, the significance of this phenomenon is not fully clear because intracellular *E. coli* did not have characteristic pathogenic features in patients with Crohn’s disease [22]. The real significance of this possible danger should be revealed in clinical trials. We have conducted preliminary acute toxicity investigation of substance N3 (liquid form of DIM) in rats, which confirmed high safety of this formulation (LD_50_ > 5,000 mg/kg). According to histological studies, acute intragastric administration of our formulation to rats at high doses (up to 5,000 mg/kg) did not cause any damage in their internal organs, particularly in mucous membrane of the stomach and intestine (data not shown). This indicates a low toxicity of this liquid DIM formulation. We plan for further preclinical and clinical trials of safety and efficacy of the liquid DIM formulation.

## Abbreviations

AUC: Area under the curve; AUP: Area under the peak; BRCA1 gene: Breast cancer susceptibility 1 gene; Cmax: the maximum drug concentration in blood plasma; CV: Coefficient of variation; DIM: 3,3′-Diindolylmethane; ERα: Estrogen receptor α; HPLC: High-performance liquid chromatography; I3C: Indole-3-carbinol; MRT: The mean residence time of drug in the body; SDM: Standard deviation of the mean; T1/2: Drug elimination half-life; Vss: The apparent steady-state volume of drug distribution.

## Competing interests

The authors declare that they have no competing interests.

## Authors’ contributions

MP participated in the design and coordination of the study. VK participated in the design and coordination of the study. EM participated in the design of the study and drafted the manuscript. VD carried out the pharmacokinetic studies and drafted the manuscript. IK carried out the pharmacokinetic studies and drafted the manuscript. OP carried out the pharmacokinetic studies and drafted the manuscript. All authors read and approved the final manuscript.
